# Development of a predictive risk stratification tool to identify the population over age 45 at risk for new-onset stroke within 7 years

**DOI:** 10.3389/fnagi.2023.1101867

**Published:** 2023-06-14

**Authors:** Kang Yang, Minfang Chen, Yaoling Wang, Gege Jiang, Niuniu Hou, Liping Wang, Kai Wen, Wei Li

**Affiliations:** ^1^Department of Geriatrics, Union Hospital, Tongji Medical College, Huazhong University of Science and Technology, Wuhan, China; ^2^Department of Thyroid, Breast, and Vascular Surgery, Xijing Hospital, Fourth Military Medical University, Xi'an, China; ^3^School of Software and Microelectronics, Peking University, Beijing, China

**Keywords:** new-onset stroke, prediction, nomogram, triglyceride-glucose index, low physical performance

## Abstract

**Background and purpose:**

With the acceleration of the aging process of society, stroke has become a major health problem in the middle-aged and elderly population. A number of new stroke risk factors have been recently found. It is necessary to develop a predictive risk stratification tool using multidimensional risk factors to identify people at high risk for stroke.

**Methods:**

The study included 5,844 people (age ≥ 45 years) who participated in the China Health and Retirement Longitudinal Study in 2011 and its follow-up up to 2018. The population samples were divided into training set and validation set according to 1:1. A LASSO Cox screening was performed to identify the predictors of new-onset stroke. A nomogram was developed, and the population was stratified according to the score calculated through the X-tile program. Internal and external verifications of the nomogram were performed by ROC and calibration curves, and the Kaplan-Meier method was applied to identify the performance of the risk stratification system.

**Results:**

The LASSO Cox regression screened out 13 candidate predictors from 50 risk factors. Finally, nine predictors, including low physical performance and the triglyceride-glucose index, were included in the nomogram. The nomogram's overall performance was good in both internal and external validations (AUCs at 3-, 5-, and 7-year periods were 0.71, 0.71, and 0.71 in the training set and 0.67, 0.65, and 0.66 in the validation set, respectively). The nomogram was proven to excellently discriminate between the low-, moderate-, and high-risk groups, with a prevalence of 7-year new-onset stroke of 3.36, 8.32, and 20.13%, respectively (*P* < 0.001).

**Conclusion:**

This research developed a clinical predictive risk stratification tool that can effectively identify the different risks of new-onset stroke in 7 years in the middle-aged and elderly Chinese population.

## 1. Introduction

The prevalence and mortality of stroke are increasing rapidly around the world. Stroke deaths per 100,000 population increased from 2,298 in 1990 to 2,633 in 2017, i.e., an increase of 14.6% (Zhou et al., 2019). Stroke has become one of the top 10 causes of death worldwide (GBD 2016 Causes of Death Collaborators, 2017) among the elderly population. At present, the number of stroke patients in China is more than 10 million, and the annual growth rate is 8.7% (Wang Y.-J. et al., 2020). The acceleration of the aging process in China poses great challenges in reducing stroke occurrence, morbidity, and mortality. Accurately identifying people at high risk of stroke and implementing precise management are essential for strengthening public health and reducing social burden.

In addition to the widely recognized classic factors for stroke risk, such as hypertension, diabetes, obesity, sex, low-density lipoprotein C, and atrial fibrillation (Xia et al., 2019; Wang Y.-J. et al., 2020), serum markers (Singer et al., 2019) (e.g., advanced glycation end products, C-reactive protein, serum homocysteine, amino acids, and sex hormones) and social factors, such as education, sports activities, region, and income (Qi et al., 2020) were found to be associated with an increased risk of stroke. In particular, the triglyceride-glucose index (TyG) (Guerrero-Romero et al., 2010; Zhao et al., 2021) and atherogenic index of plasma (AIP) (Pepe et al., 2020; Wang C. et al., 2020) were found to be closely associated with stroke. There is a significant linear relationship between AIP and stroke (Ding et al., 2019), and TyG, which represents insulin resistance, is associated with stroke recurrence and increases the risk of all-cause mortality (Li et al., 2018). Although new risk factors for stroke are constantly being discovered and confirmed, the predictors included in the existing stroke prediction models are always traditional factors, with or without screening. Therefore, it is very necessary to develop a new stroke prediction model involving risk factors over a wide range and multiple dimensions as the candidate variables.

The study of the stroke risk prediction model was originally developed using the Western population (Chambless et al., 2004; Hippisley-Cox et al., 2013; Dufouil et al., 2017). Therefore, these prediction models may not be suitable for the Chinese population due to the differences in race, lifestyle, stroke morbidity, and so on. Among the currently developed predictive models, there are stroke predictors in specific populations [diabetes (Li et al., 2018; Shi et al., 2020) and atrial fibrillation (Menon et al., 2012)] or predictors of adverse outcomes after stroke (cognitive dysfunction, mortality, depression, etc.). However, a few predictors of new-onset stroke have been observed in the middle-aged and elderly Chinese population during long-term longitudinal follow-up.

By incorporating a number of traditional, nontraditional, and newly identified stroke risk factors through the reliable screening process, this study has developed a 7-year prediction model, further stratified Chinese residents over 45 years into low-, moderate-, and high-risk groups for new-onset stroke, and conducted external validation.

## 2. Methods

### 2.1. Population

The China Health and Retirement Longitudinal Study (CHARLS) is a survey of middle-aged and elderly people in China. The subjects were randomly selected from 450 villages in 28 provinces and 150 counties and districts across the country among those aged 45 years and older (Zhao et al., 2014). CHARLS adopts a multistage, stratified, and proportional sampling method with probability and scale. The baseline study started in 2011, followed by sampling every 2 years (2011: wave1, 2013: wave2, 2015: wave3, and 2018: wave4). The baseline data included assessments of social-related issues, economic-related conditions, and health status for 17,708 participants using face-to-face interviews and questionnaires (Zhao et al., 2013).

The use of CHARLS was approved by the Ethics Committee of Yang et Peking University Health Science Center and obtained informed consent from all participants.

**Figure 1:**
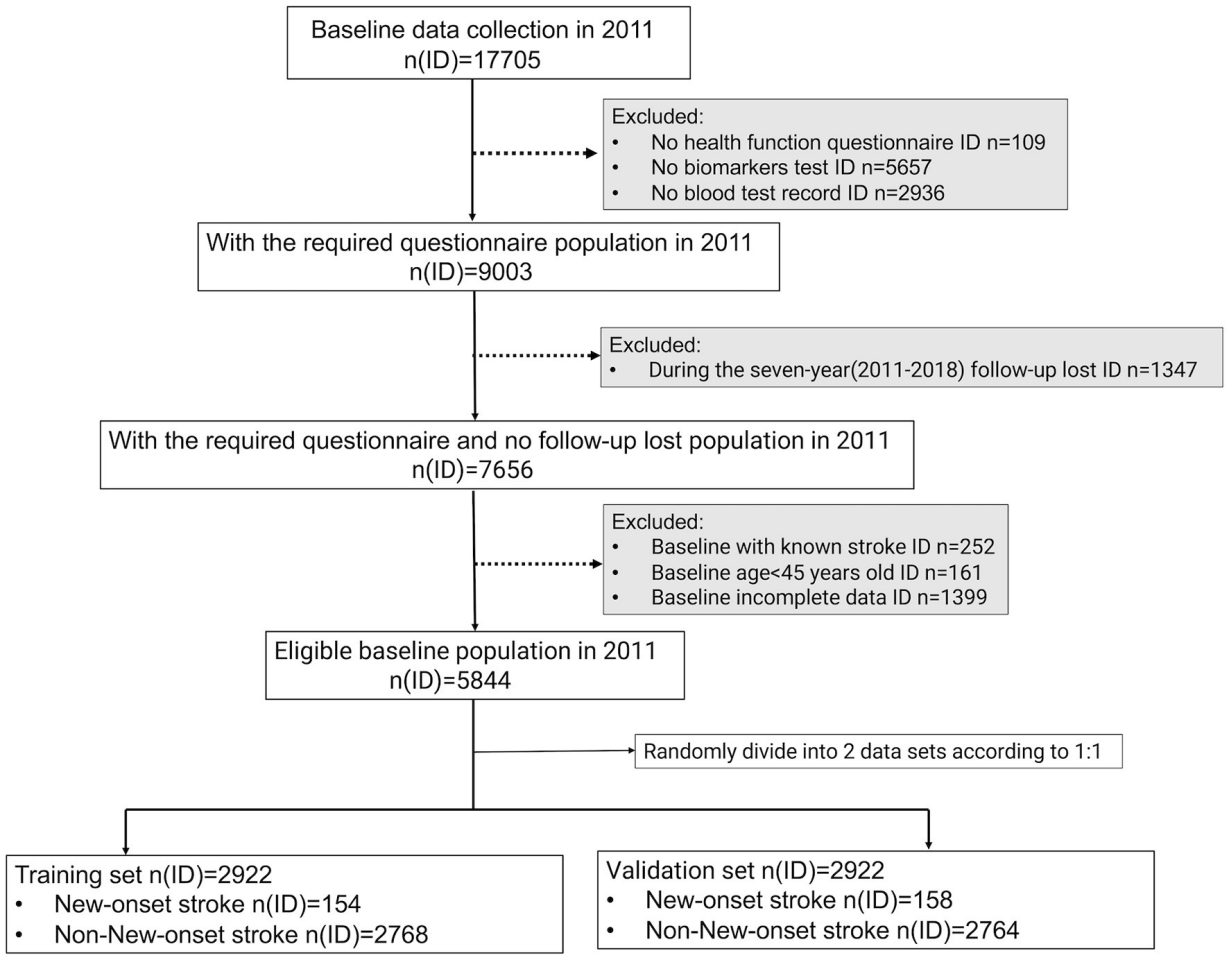
Flowchart of the study design. To conduct follow-up research, the physical examination, household questionnaire information, and blood test data information of eligible participants must be accessible simultaneously. In addition, they must be over 45 years old, with no history of stroke at baseline, and not be missing a 7-year follow-up. In the end, 5,844 participants were eligible to be included in the analysis of this study and were randomly assigned to the training and validation sets in a ratio of 1:1.

The flowchart details of the study design are shown in Figure 1.

### 2.2. Outcome indicators and candidate predictors

#### 2.2.1. Outcome indicators

Outcome indicators included whether a stroke occurred during follow-up (new-onset stroke: yes/no) and how long (time: in years) has elapsed since baseline. All individuals were interviewed for the first time in 2011 and last interviewed in 2018. The detailed compilation process is available in the Supplementary material.

#### 2.2.2. Candidate predictors

According to epidemiological characteristics and previous studies, a total of 55 risk factors were included in three aspects.

(A) Physical examination index: age, sex, systolic blood pressure (SBP), diastolic blood pressure (DBP), pulse pressure (PP), body mass index (BMI), waist circumference (WC), and handgrip strength were included in the study. The BMI group was divided into the following four subgroups according to the Chinese standards: low weight (BMI < 18.5 kg/m^2^), normal weight (18.5 kg/m^2^ ≤ BMI < 24.0 kg/m^2^), overweight (24.0 kg/m^2^ ≤ BMI < 28.0 kg/m^2^), and obesity (BMI ≥ 28.0 kg/m^2^) (Fu et al., 2018).

(B) Health status and function test:

(a) Cognition and depression: The total cognition score was defined as the sum of the scores for episodic memory, telephone interview of cognitive status (TICS), and painting ability. CHARLS uses episodic memory and executive function to evaluate participants' cognition. Episodic memory (0–10) includes immediate memory (0–10) and delayed memory (0–10). Executive function was evaluated through TICS (0–10) and painting ability (0–1). TICS was used as a Mini-Mental State Examination to screen for cognitive impairment in the elderly population (Wei et al., 2018; Lök et al., 2019), including orientation (0–5) and calculation (0–5). The 10-item Center for Epidemiological Studies Depression Scale (CESD-10; Chen and Mui, 2014) was used to assess depression. The score range is 0–30.
(b) Sleep duration: Nap and nighttime sleep duration were recorded separately to find the length of naps (minutes) and the length of night sleep (hours) in the past month (Li et al., 2017).
(c) Functional status: This indicator was measured by activities of daily living (ADL, 0–6; Katz et al., 1963) and instrumental activities of daily living (IADL, 0–5; Lawton and Brody, 1969).
(d) Social and intellectual activities (Li et al., 2020): Scores were assigned according to the frequencies of activities (almost daily = 3, almost every week = 2, and not regularly = 1), and the total scores of each item were calculated for social and intellectual activities, respectively.
(e) Physical performance (Chen et al., 2020): The chair stand test was used to evaluate physical performance.
(f) History of disease and smoking or alcohol use: The 12-disease history (yes/no) includes hypertension (HTN), dyslipidemia, diabetes or high blood sugar, chronic lung disease, liver disease, heart disease, kidney disease, stomach or other digestive diseases, memory-related disease, emotional, nervous, or psychiatric problems (ENP), arthritis or rheumatism (Arth/Rheu), and asthma. Similarly, a history of smoking (yes/no) and alcohol use (often/sometimes/never) were also used in the study.

(C) Blood test indicators and composite indicators: The blood test indicators used are as follows: white blood cells (WBC), hemoglobin (Hgb), platelet counts (PLT), hematocrit (HCT), mean corpuscular volume (MCV), high-sensitivity C reactive protein (hs-CRP), glycosylated hemoglobin (HbA1c), HDLC, LDLC, cholesterol (CHOL), triglycerides (TG), glucose (GLU), blood urea nitrogen (BUN), creatinine (Crea), and uric acid (UA). The composite indicators used include the triglyceride-glucose index (TyG) = ln [TG (mg/dL) ×GLU (mg/dL)/2] (Guerrero-Romero et al., 2010; Zhao et al., 2021) and atherogenic index of plasma (AIP) = ln [TG (mg/dL)/HDL (mg/dL)] (Wang C. et al., 2020).

More detailed information on all indicators is provided in the Supplementary material.

### 2.3. Statistical methods

With equal probability random sampling, we allocated the final sample (5,844) into two groups, namely, the training set (2,922) and the validation set (2,922), in a theoretical ratio of 1:1 (Call the function “sample” in the R, the detailed code could be available in Supplementary material). Comparisons of differences between groups were analyzed using the ANOVA test and the Kruskal–Wallis test for continuous variables with normal and non-normal distributions, respectively, and using the chi-squared test for categorical variables.

Considering the number, wide latitude, and possible collinearity of the candidate variables before the screening, we first adopted the least absolute shrinkage and selection operator (LASSO) Cox regression method to reduce the data dimension and avoid overfitting (R package:glmnet). By constructing an L1 penalty function to obtain a refined model, the LASSO could filter out some variables with coefficients of 0. By Cox multiple regression analysis on the selected variables using the backward method, we obtain the hazard ratio (HR) and the 95% confidence interval (95% CI) of the selected variables and determine the independent predictors for the 7-year new-onset stroke. A nomogram was constructed according to the final multivariate Cox regression analysis model, and the 3-, 5-, and 7-year risk points for new-onset stroke were calculated for every individual in the training and validation sets, respectively. The ROCs and calibration curves (bootstrap method = 1,000) were plotted to evaluate the predictive performance of the nomogram for the 3-, 5-, and 7-year new-onset stroke incidents according to the risk score in both sets. We used the X-tile software (version 3.6.1; Yale, New Haven, USA) to determine the optimal cutoff value of the risk points of the nomogram model and stratified the population into low-, moderate-, and high-risk groups for new-onset stroke incidents based on this cutoff value. The Kaplan–Meier method and the log-rank tests were performed to identify the cumulative incidence in different risk groups and compare the difference between groups, respectively. A *P*-value of < 0.05 was considered to be statistically significant. All calculations and graphs were done using R (version 3.6.3-Mac OS X 10.11).

## 3. Results

### 3.1. Baseline characteristics in the training and validation sets

Using a randomization procedure, we separately allocated 2,922 samples in the training and validation sets. A total of 154 and 158 new-onset stroke incidents occurred in the training and validation sets, respectively, during the 7-year follow-up. Except for BMI (*P* = 0.05), WC (*P* = 0.002), nighttime sleep duration (*P* = 0.041), and history of chronic lung diseases (*P* = 0.047), the remaining 49 variables did not show significant differences between the groups, revealing that the training and validation sets were homogeneous in almost all the dependent variables (Supplementary Table 1).

### 3.2. Baseline characteristics of the training set stratified by the occurrence of a new-onset stroke

The baseline average age of people with new-onset stroke events during follow-up was significantly greater than that of those without new-onset stroke events (61.25 vs. 58.12). There was no significant difference in the baseline sex ratio. The proportion of overweight (26.6 vs. 26.4%) and obese (7.1 vs. 4.4%) people in the cohort of subjects with new-onset stroke events was significantly higher than that of those in the cohort without new-onset stroke events. The people who suffered stroke incidents, compared with those who did not, had significantly higher average SBP (141.08 vs. 128.56 mmHg), DBP (79.67 vs. 75.21 mmHg), and PP (73.90 vs. 72.03 mmHg) levels at baseline. People who suffered a new-onset stroke during the follow-up generally had lower baseline cognitive scores than those who did not have a stroke; however, the depression score (CESD-10) was the opposite. However, those differences did not reach statistical significance. In particular, the proportion of people with new-onset stroke during follow-up who had a lower physical performance at baseline was significantly higher than that of those without stroke (39.0 vs. 27.1%, *p* = 0.002).

By comparing the baseline medical histories of the two groups of people, in addition to the traditional stroke risk factors such as hypertension (34.4 vs. 20.4%), dyslipidemia (17.5 vs. 9.1%), heart disease (16.2 vs. 9.8%), diabetes, or high blood glucose (11.0 vs. 5.2%), people with a history of ENP (5.2 vs. 1.9%) or memory-related disease (3.2 vs. 0.9%) were also found to have significantly higher proportions of new-onset stroke events during follow-up.

Regarding blood indicators between groups, the baseline TG (122.57 vs. 101.78), CHOL (196.97 vs. 190.21), blood glucose (106.02 vs. 101.88), and C-reactive protein levels (1.32 vs. 0.97) of the group with new-onset stroke were higher than those of the group without new-onset stroke. The composite indicators TyG (4.72 vs. 4.63) and AIP (0.85 vs. 0.71) also display similar significant intergroup differences (Table 1).

**Table 1:** Baseline characteristics of the training set participants stratified according to new-onset stroke at 7-year follow-up.

**Characteristics^†^**	**Stroke free**	**New-onset stroke**	***P* ^‡^**
***n***	**2,768**	**154**	
Age	58.12 (8.51)	61.25 (8.93)	< 0.001
Gender = men (%)	1,299 (46.9)	69 (44.8)	0.666
**BMI-group** =			
Low (%)	192 (6.9)	7 (4.5)	0.294
Normal (%)	1,725 (62.3)	95 (61.7)	
Overweight (%)	730 (26.4)	41 (26.6)	
Obese (%)	121 (4.4)	11 (7.1)	
SBP (mmHg)	128.56 (20.44)	141.08 (25.62)	< 0.001
DBP (mmHg)	75.21 (11.94)	79.67 (12.75)	< 0.001
PP (mmHg)	72.03 (10.17)	73.90 (10.04)	0.026
WC (cm)	83.59 (12.66)	84.44 (16.59)	0.426
Handgrip strength (kg)	30.61 (14.22)	29.60 (9.73)	0.386
Total cognition score	10.55 (4.21)	10.00 (3.94)	0.116
TICS	6.58 (2.83)	6.31 (2.79)	0.241
Orientation	3.74 (1.40)	3.61 (1.47)	0.255
Computation	2.84 (1.98)	2.69 (2.00)	0.388
Drawing ability	0.64 (0.48)	0.60 (0.49)	0.306
Episodic memory	3.33 (1.89)	3.09 (1.71)	0.137
Immediate memory	3.76 (1.97)	3.63 (1.75)	0.413
Delayed memory	2.89 (2.05)	2.56 (1.93)	0.052
CESD-10	9.84 (4.87)	10.25 (5.09)	0.305
Nighttime sleep duration (h)	6.34 (1.86)	6.18 (2.05)	0.292
Napping duration (min)	31.73 (43.02)	36.21 (46.01)	0.209
IADL	0.30 (0.79)	0.36 (0.79)	0.413
ADL	2.42 (1.40)	2.36 (1.33)	0.626
Social activity	1.06 (1.55)	1.14 (1.62)	0.548
Intellectual activity	0.40 (0.90)	0.38 (0.85)	0.798
Smoke = yes (%)	1,095 (39.6)	65 (42.2)	0.569
**Alcohol use** =			
Often (%)	732 (26.4)	46 (29.9)	0.608
Sometimes (%)	210 (7.6)	10 (6.5)	
Never (%)	1,826 (66.0)	98 (63.6)	
Physical performance = low (%)	751 (27.1)	60 (39.0)	0.002
**Medical history** =			
Hypertension (%)	564 (20.4)	53 (34.4)	< 0.001
Dyslipidemia (%)	253 (9.1)	27 (17.5)	0.001
Lung disease (%)	273 (9.9)	21 (13.6)	0.168
Liver disease (%)	120 (4.3)	7 (4.5)	1
Heart disease (%)	272 (9.8)	25 (16.2)	0.015
Kidney disease (%)	166 (6.0)	10 (6.5)	0.938
DM/HGlu (%)	143 (5.2)	17 (11.0)	0.003
Digestive disease (%)	574 (20.7)	36 (23.4)	0.495
ENP (%)	52 (1.9)	8 (5.2)	0.011
Memory disease (%)	26 (0.9)	5 (3.2)	0.021
Arth/Rheu (%)	813 (29.4)	48 (31.2)	0.7
Asthma (%)	108 (3.9)	7 (4.5)	0.852
**Blood indices**			
WBC (G/L)	5.90 (4.90, 7.20)	5.85 (5.00, 7.23)	0.697
Hgb (g/dL)	14.20 (13.08, 15.50)	14.50 (13.03, 15.67)	0.737
MCV (fl)	91.10 (86.80, 95.50)	90.70 (86.22, 96.20)	0.817
PLT (G/L)	207.00 (161.00, 254.00)	209.00 (160.25, 263.75)	0.447
Crea (mg/dL)	0.76 (0.64, 0.88)	0.77 (0.68, 0.88)	0.16
BUN (mg/dL)	15.15 (12.69, 18.01)	15.04 (12.69, 17.45)	0.751
UA (mg/dL)	4.22 (3.53, 5.04)	4.39 (3.59, 5.49)	0.063
HbA1c (%)	5.10 (4.90, 5.40)	5.10 (4.90, 5.40)	0.914
hs-CRP (mg/dL)	0.97 (0.52, 1.97)	1.32 (0.63, 2.72)	0.002
LDLC (mg/dL)	114.05 (93.17, 136.86)	118.69 (96.84, 139.56)	0.146
CHOL (mg/dL)	190.21 (167.40, 215.05)	196.97 (172.62, 221.91)	0.025
Glu (mg/dL)	101.88 (94.32, 111.96)	106.02 (95.67, 118.04)	0.007
TG (mg/dL)	101.78 (73.46, 149.57)	122.57 (85.18, 169.70)	0.001
HDLC (mg/dL)	49.87 (40.98, 60.70)	47.55 (40.59, 57.51)	0.109
TyG	4.63 (4.45, 4.85)	4.72 (4.53, 4.92)	0.001
AIP	0.71 (0.24, 1.23)	0.85 (0.47, 1.43)	0.004

† Quantitative data of normal and non-normal distribution are expressed as mean ± SD and median with interquartile range (IQR). Categorical data are presented as amounts with percentages.

‡ Comparisons of differences between groups are analyzed by ANOVA test and Kruskal-Wallis test for continuous variables with normal and non-normal distribution, respectively, and by chi-squared test for categorical variables.

BMI, body mass index; SBP, systolic blood pressure; DBP, diastolic blood pressure; PP, pulse pressure; WC, waist circumference; TICS, telephone interview of cognitive status; CESD-10, the 10-item center for epidemiological studies; IADL, instrumental activities of daily living; ADL, activities of daily living; DM/HGlu, diabetes or high blood sugar; ENP, emotional, nervous, or psychiatric problems, arth/rheu arthritis or rheumatism; WBC, white blood cells; Hgb, hemoglobin; MCV, mean corpuscular; PLT, platelet counts; Crea, creatinine; BUN, blood urea nitrogen; UA, uric acid; hba1c, glycosylated hemoglobin; hs-CRP, high-sensitivity C reactive protein; LDLC, low-density lipoprotein cholesterol; CHOL, cholesterol; Glu, glucose; TG, triglycerides; HDLC, high-density lipoprotein cholesterol; TyG, triglyceride-glucose index; AIP, atherogenic index of plasma.

### 3.3. Independent prognostic factors in the training set and establishment of a prediction nomogram

**Figure 2:**
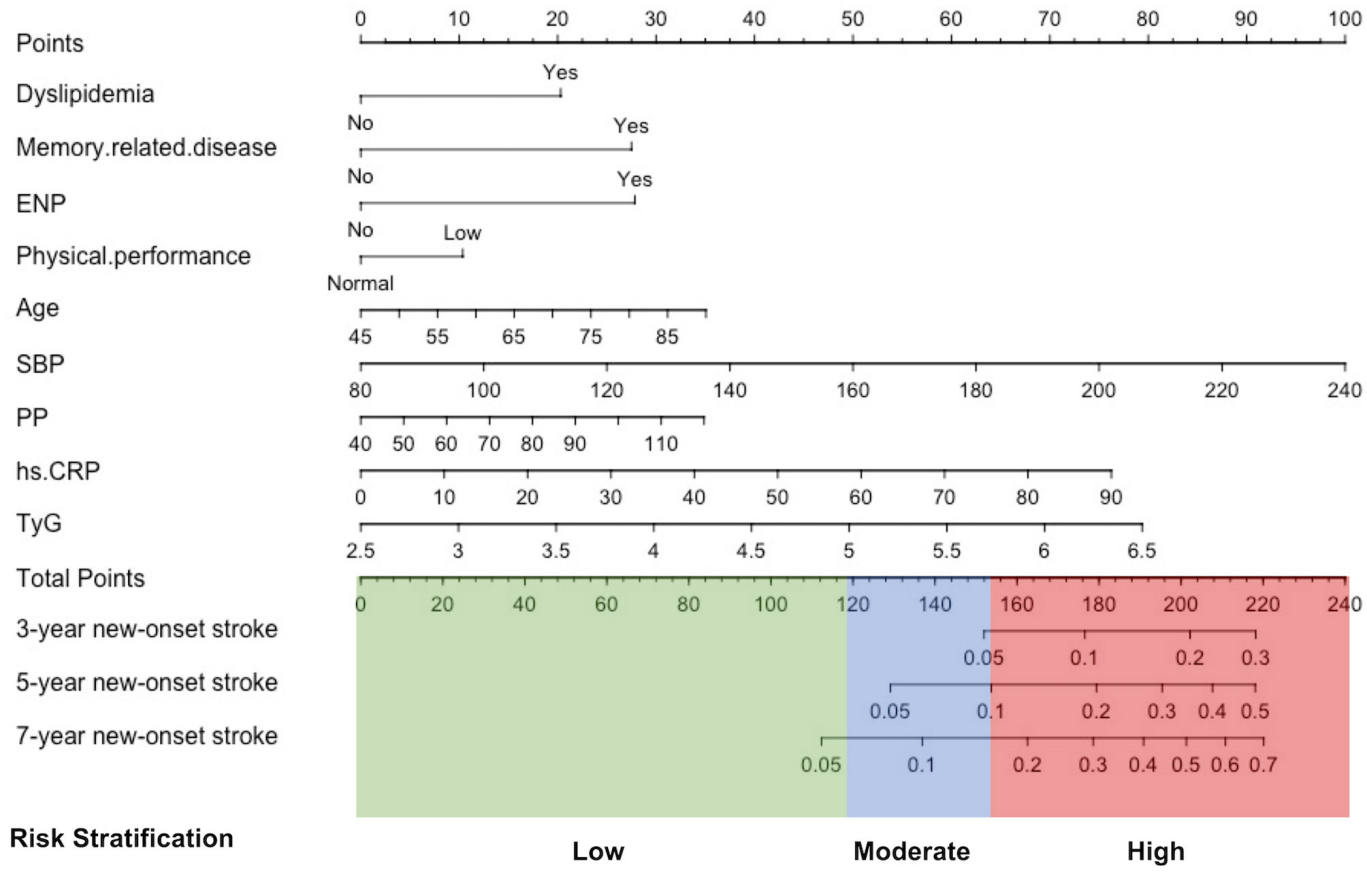
Development of a stratification nomogram for the new-onset stroke in 7 years. ENP, history of emotional, nervous, or psychiatric problems; SBP, systolic blood pressure; PP, pulse pressure; hs-CRP, high-sensitivity C reactive protein; TyG, triglyceride-glucose index.

The rates of new-onset stroke during the 3-, 5-, and 7-year periods in the training set were 1.71, 3.29, and 5.27%, respectively. 13 with non-zero coefficients were screened in the LASSO Cox regression model based on the optimal value of lambda (λ) (Supplementary Figure 2; Supplementary Table 2). Table 2 (Model 2) summarizes the results of the multiple Cox regression tests. Age, SBP, PP, Phy-G, CRP, TyG, history of dyslipidemia, ENP, and memory disease were the nine independent predictors for new-onset stroke. Table 2 (Model 1) presents the hazard ratio of these nine predictors for the outcome incident obtained by the univariable Cox regression analysis (the results of the remaining variables are available in the Supplementary material). A predictive nomogram integrating all nine independent variables was developed for predicting 3-, 5-, and 7-year new-onset stroke events (Figure 2).

**Table 2:** The hazard ratio (HR) and 95% confidence interval (95% CI) of the variables selected from the LASSO model for 7-year new-onset stroke by Cox univariable and multivariable analyses.

**Variables**	**Univariable analysis**			**Multivariable analysis** ^**†**^		
	**HR**	**95% CI**	***p***	**HR**	**95% CI**	***p***
Age	1.04	(1.022, 1.058)	< 0.01	1.023	(1.0041–1.042)	0.017
SBP	1.023	(1.017, 1.030)	< 0.01	1.018	(0.9979–1.028)	< 0.01
PP	1.017	(1.002, 1.032)	0.026	1.013	(0.9979–1.028)	0.093
Low physical performance	0.591	(0.423, 0.817)	< 0.01	1.354	(0.9695–1.891)	0.075
Dyslipidemia	2.066	(1.363, 3.129)	< 0.01	1.812	(1.186–2.768)	0.006
Memory disorders	3.384	(1.388, 8.249)	< 0.01	2.236	(0.8717–5.737)	0.094
ENP	2.801	(1.375, 5.707)	< 0.01	2.259	(1.0719–4.76)	0.032
CRP	1.029	(1.015, 1.044)	< 0.01	1.025	(1.0103–1.04)	< 0.01
TyG	2.335	(1.502, 3.630)	< 0.01	1.787	(1.1099–2.879)	0.017

BMI, body mass index; SBP, systolic blood pressure; DBP, diastolic blood pressure; PP, pulse pressure; WC, waist circumference; TICS, telephone interview of cognitive status; CESD-10, the 10-item center for epidemiological studies; IADL, instrumental activities of daily living; ADL, activities of daily living; DM/HGlu, diabetes or high blood sugar; ENP, emotional, nervous, or psychiatric problems, arth/rheu arthritis or rheumatism; WBC, white blood cells; Hgb, hemoglobin; MCV, mean corpuscular; PLT, platelet counts; Crea, creatinine; BUN, blood urea nitrogen; UA, uric acid; hba1c, glycosylated hemoglobin; hs-CRP, high-sensitivity C reactive protein; LDLC, low-density lipoprotein cholesterol; CHOL, cholesterol; Glu, glucose; TG, triglycerides; HDLC, high-density lipoprotein cholesterol; TyG, triglyceride-glucose index; AIP, atherogenic index of plasma.

† Considering the simplicity and practicality of the nomogram as a clinical assessment tool, we applied Cox regression using the backward method to further optimize the model. The initial variables included were 13 variables from the LASSO regression model, and finally, nine variables were obtained as the constituent factors of the nomogram for predicting a 7-year new-onset stroke.

### 3.4. Validation of the nomogram performance

**Figure 3:**
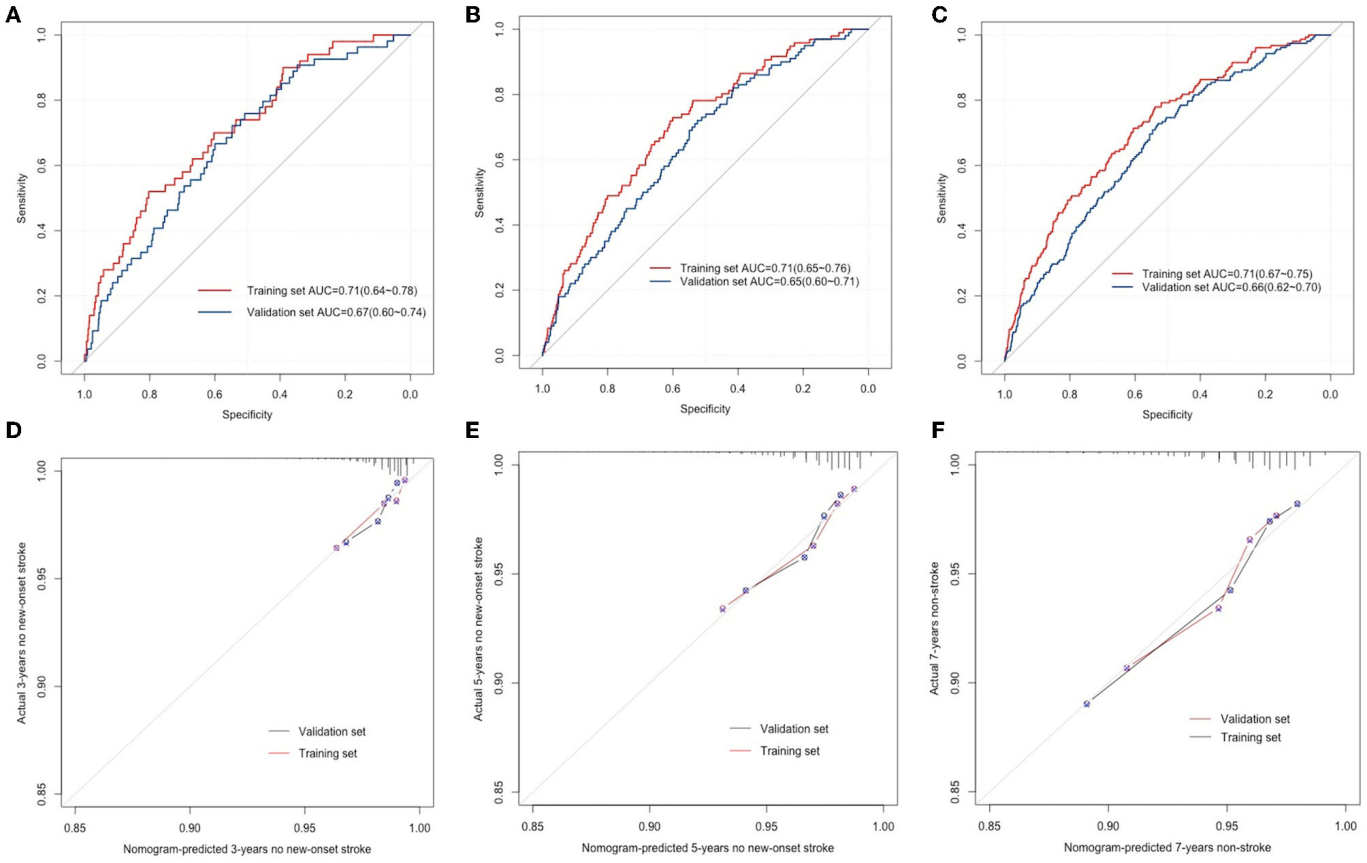
ROC curves for the prediction of new-onset stroke in the training and validation sets at 3 years **(A)**, 5 years **(B)**, and 7 years **(C)**, respectively. The calibration curves for predicting the new-onset stroke population in the training and validation sets at 3 years **(D)**, 5 years **(E)**, and 7 years **(F)**, respectively.

To validate the differentiation performance of the nomogram with the outcome incidents, ROCs were created for the model to discriminate new-onset stroke events at 3, 5, and 7 years of follow-up. In the training cohort, the areas under the ROC curve (AUCs) for the prediction of outcome at the 3-, 5-, and 7-year time points were 0.71 (95% CI, 0.64–0.78), 0.71 (95% CI, 0.65–0.76), and 0.71 (95% CI, 0.67–0.75). Similarly, the AUCs in the validation were 0.67 (95% CI, 0.60–0.74), 0.65 (95% CI, 0.60–0.71), and 0.66 (95% CI, 0.62–0.70), respectively (Figures 3A–C).

The developed nomogram was used to predict the total points and probability of new-onset stroke incidents for everyone in the training and validation sets. The predicted probability was compared with the actual occurrence rate to evaluate the predictive performance of the model. The 3-, 5-, and 7-year calibration curves displayed the developed nomogram's superior agreement between predictions and the actual occurrence rate for stroke incidents in follow-up (Figures 3D–F).

### 3.5. Development of a prediction stratification model

The 2,922 individuals in the training set were stratified into three risk groups using the X-tile program according to individuals' total points calculated by nomogram: low risk (2,174 individuals; total points ≤ 119.2); moderate risk (589 individuals; 119.2 < total points ≤ 146.8); and high risk (159 individuals; total points > 146.8). The survival curves showed excellent discrimination for 7-year stroke probabilities when the nomogram total points were categorized into low-, moderate-, and high-risk groups by X-Tile, with 7-year new-onset stroke probability of 3.36, 8.32, and 20.13%, respectively (*P* < 0.001, Supplementary Figure 2). The same point threshold was utilized to stratify the population in the validation set into groups of low risk (2,130 individuals), moderate risk (624 individuals), and high risk (168 individuals). The survival curve presents similar good discrimination in the validation set for the 7-year stroke probability (*P* < 0.001, Supplementary Figure 2), with the 7-year new-onset stroke cumulative incidence rates of 4.18, 7.69, and 12.50%, respectively. Compared with the low-risk group, the hazard ratio of the prediction stratification model in the training set was higher in the moderate-risk group (2.54, 95% CI: 1.77–3.66) and the high-risk group (6.60, 95% CI: 4.36–10.00). In the validation set, that was the moderate-risk group (1.87, 95% CI: 1.32–2.66) and the high-risk group (3.12, 95% CI: 1.94–5.02) separately (Table 3).

**Table 3:** The hazard ratio (HR) and 95% confidence interval (95% CI) of different risk groups stratified by the prediction stratification model for new stroke events in 7 years in the whole cohort, training set, and validation set.

	**Overall**			**Training set**				**Validation set**				
**Risk stratification**	***n***	**HR**	**95% CI**	***P***	***n***	**HR**	**95% CI**	***P***	***n***	**HR**	**95% CI**	***P***
Low	4,304 (162)				2,174 (73)	–	–	–	2,130 (89)	–	–	–
Moderate	1,213 (97)	2.17	(1.69–2.79)	< 0.001	589 (49)	2.537	(1.77, 3.66)	< 0.001	624 (48)	1.873	(1.32, 2.66)	< 0.001
High	327 (53)	4.61	(3.38–6.23)	< 0.001	159 (32)	6.600	(4.36, 10.00)	< 0.001	168 (21)	3.119	(1.94, 5.02)	< 0.001

## 4. Discussion

We screened out nine independent risk factors from 50 potential risk contributors and developed a predictive stratification model with them to identify new-onset stroke probability in low-risk, moderate-risk, and high-risk groups among the middle-aged and elderly population (≥45) based on a large sample population across the country from CHARLS. After being thoroughly validated internally and externally, the model showed good performance in predicting 3-, 5-, and 7-year new-onset stroke incidents and determining the different risk individuals, which provides an effective clinical tool to identify potential risk groups and administer modifiable risk factors to these people.

In this study, age, SBP, PP, CRP, TyG, physical performance, history of dyslipidemia, memory-related disease, and ENP problems were independent predictors for new-onset stroke. Consistent with previous studies, age, SBP, PP, CRP, and dyslipidemia play important roles in stroke. In addition, our study demonstrated that patients with low physical performance and a history of memory-related diseases and ENP problems are usually at a high risk of new-onset stroke. Studies have shown that physical performance and ENP problems are predictors of stroke (McGinn et al., 2008) and important indicators of stroke prognosis (Maeda et al., 2000; Cohen et al., 2018; McCurley et al., 2019). However, to date, there has been no study to incorporate them into the stroke prediction models but we did that in this study. Interestingly, although the baseline memory score was not considered an independent risk factor for new-onset stroke in this study, which was a controversial opinion in previous research (Wang et al., 2012, 2014; Rostamian et al., 2015), the history of memory-related diseases, retained as an independent predictor and assigned a high-risk prediction score, was included in the final predictive model. This demonstrates that the history of memory-related diseases may be more reliable than memory-related cognitive tests in predicting new-onset strokes. In particular, TyG, a simple surrogate marker related to insulin resistance (Guerrero-Romero et al., 2010), has been confirmed to be closely related to stroke incidence in some regional studies. In this nationwide longitudinal tracking cohort, TyG was found to be independently associated with new-onset stroke during 7-years of follow-ups (HR 1.79, 95% CI 1.11–2.88) in the multivariable model and was included for the first time in the new-onset stroke prediction nomogram.

We included all of these risk factors in the predictive stratification model. Our results show that the model can well-identify the high-risk population for stroke in 7 years. Doctors may provide timely clinical intervention to potentially high-risk patients to enable them to benefit from the assessment. Different stroke prediction models based on demographic information and clinical measurements have been developed in Europe, North America (Chambless et al., 2004; Hippisley-Cox et al., 2013), and Asian populations (Jee et al., 2008). However, due to racial and environmental differences, these models do not perform well in other populations. For example, the Framingham model developed by Wolf et al. (1991), although widely used in the United States, France, and other regions, is not applicable to China because it overestimates the risk of coronary heart disease in the Chinese population (Liu et al., 2004). This finding emphasizes the necessity of developing risk prediction models for the Chinese population, which is consistent with the concepts of the American Heart Association (Lloyd-Jones et al., 2019). For Chinese people, some models have been developed. In 2011, Gan et al. (2011) established a classification tree model for stroke prediction in a southern China hospital that included risk factors such as physical exercise, history of hypertension, tea drinking, HDL-c level, smoking status, and educational level. The AUC was 0.79. In 2016, Wang et al. (2016) developed a life-long stroke risk map and risk chart in a Chinese multiprovincial cohort study for the young and middle-aged population, containing six traditional risk factors (blood pressure, non-HDL-C, HDL-C, BMI, diabetes, and smoking), but lacking external verification. In 2020, Chien et al. developed a 10-year stroke risk prediction model from the community cohort of 3,513 Taiwan participants in China, including seven variables: age, gender, systolic and diastolic blood pressure, family history of stroke, atrial fibrillation, and diabetes mellitus. The AUC was 0.772 (95% CI, 0.744–0.799) (Chien et al., 2010). However, the tool still lacks external verification, and the nomogram predicts stroke-free probability instead of new-onset stroke risk, which makes that tool inconvenient to use in clinical scenarios. Another 2-year new stroke risk prediction model was developed using logistic regression based on Chinese people aged above 45 years, including five risk factors, namely, heart disease, hypertension status, age, diabetes, and smoking (Yao et al., 2020). In total, these models have certain limitations in the region and period, with or without external verification, and most of the variables included in the models were screened from traditional risk factors, such as age, gender, BMI, diabetes, and blood pressure, or without screening, which is detrimental to the development of predictive models.

In this nomogram for the prediction of new-onset stroke, the sample cohort from CHARLS, a nationally representative survey, was followed up for up to 7 years. The 3-, 5-, and 7-year new-onset stroke probabilities were predicted, which showed excellent representation in time and space. In addition, the number of candidate variables we included in our models was up to 50, other than just some traditional risk factors, involving cognition, depression, sleep duration, daily functional status (ADL, IADL), social and personal activities, physical performance, history of 13 chronic diseases, etc. We filtered out and evaluated the predictive value of all of them by LASSO regression and Cox regression analysis using the backward method. Thus, we paid attention to some easily overlooked risk factors for new-onset stroke, namely, memory disease, EBP problems, and low physical performance, which play significant roles in the new-onset stroke nomogram. However, some previous studies have verified TyG's risk for stroke. We confirmed that in our longitudinal cohort and integrated it into the prediction model in our first attempt. Furthermore, we calculated the risk score for everyone with our nomogram, obtained the different risk cutoff values for nomogram points using the X-Tile software, and, for the first time, classified the low-, moderate-, and high-risk groups based on the cutoff values. It is convenient for clinicians to assess stroke risk and has a certain application value in public health medical practice. The importance of these variables in predicting new-onset stroke also indicates that attention should not be focused solely on the clinical indicators or traditional risk factors but on a comprehensive assessment of the patient's health, but the psychophysical and physical performance state cannot be ignored.

For developing countries, such as China, that are characterized by a large population base, unbalanced medical and health resources, an accelerated aging process, and a heavy public health burden related to cardiovascular and cerebrovascular events, it is a very important measure to immerse preventive healthcare workers in the community.

The development of a prediction model derived from the population of medical institutions may have high prediction efficiency because each sample contains an increasingly accurate population of medical characteristics, such as more detailed blood test indicators, electrocardiogram information, and drug use history. Samples from medical institutions may be biased (higher incidence rate, a higher proportion of medical history, etc.), which may overestimate the practical application value of such models and cannot be generally applied to primary healthcare prevention work. The model in this study was derived from community surveys, which could better adapt to this census and obtain higher practicality and response rates. Even if its predictive performance is not optimal, it is of great significance to reduce the risk of stroke in the overall population, identify stroke risk groups in primary health centers or community surveys, and further remind these groups to participate in preventive healthcare or seek medical treatment.

There are also some limitations: given that the training and validation populations of this model are residents of the Chinese community, the predictive efficiency of ethnicities other than East Asians may be limited. Although we included many variables, such as candidate screening, the major variables such as a history of atrial fibrillation and a family history of stroke were lacking. Considering the significant risk of atrial fibrillation or a family history of stroke, it may improve the predictive efficiency of the model. Therefore, in the follow-up design of community cohort studies or primary health surveys, supplementing the history of atrial fibrillation or having the investigators conduct cardiac auscultation on the subjects is of great significance to evaluate the short- and long-term incidence and mortality of cardiovascular- and cerebrovascular-related events. Second, the CHARLS questionnaire did not classify stroke as intracranial hemorrhage or ischemic stroke; therefore, we did not clearly point out stroke subtypes, which may lead to different risk factors and biases.

## 5. Conclusion

We used the LASSO-Cox regression model and the X-Tile tool to develop a stroke prediction tool including nine risk factors from 50 basic population characteristics from the nationwide large-scale longitudinal community survey population and determined the low-, medium-, and high-risk classification thresholds for new-onset stroke in the short, medium, and long terms (3, 5, and 7 years). In this model, the predictive value of age, blood pressure, pulse pressure, history of hyperlipidemia, history of memory-related diseases, emotional, nervous, or psychiatric problems, low physical performance, hs-CRP, and TyG level on new stroke events are emphasized.

## Data availability statement

Publicly available datasets were analyzed in this study. This data can be found here: http://charls.pku.edu.cn/index/zh-cn.html.

## Ethics statement

CHARLS was approved by the Ethics Committee of Peking University Health Science Center and obtained informed consent from all participants. The patients/participants provided their written informed consent to participate in this study.

## Author contributions

WL: conceptualization. KY, MC, YW, and LW: data acquisition. KY, YW, and KW: statistical analysis. MC, GJ, and YW: writing–original draft. NH: statistical advice and interpretation of results. YW: writing–review and editing. All authors contributed to the article and approved the submitted version.
