# Supplementary material for: Development of a predictive risk stratification tool to identify the population over age 45 at risk for new-onset stroke within 7 years
Source: Front Aging Neurosci. 2023 Jun 14;15:1101867. doi: 10.3389/fnagi.2023.1101867 (PMC10301757; doi:10.3389/fnagi.2023.1101867)
Supplement: Supplementary file 1 [file Data_Sheet_1.docx]

Supplementary Material

# Supplementary Methods

## Outcome indicators

We integrated the answers to the code da009_1_8(“When was the condition(stroke) first diagnosed or known by yourself?”) in the CHARLS-HEALTH STATUS AND FUNCTIONING QUESTIONNAIRE at wave1(2011), wave2(2013), wave3(2015), and wave4(2018). If an individual has more than two records, the record of the most recent interview shall prevail. We considered the participants with answer≥2011 as meeting a new-onset stroke incident during the follow-up, and those with answer<2011 as having a history of stroke. In addition, we calculated Time as "stroke-year"-2011. Especially, we command Time=1 when the value range of Time is [0,1).

## Episodic memory, Immediate memory, and Delayed memory

Participants were given 10 irrelevant Chinese words, and repeated them as many as possible immediately (instant memory) and 4 minutes later (delayed memory) as required. A correct word was given one point, with a full score of 20 points. The way we calculate the episodic memory score is the average of the instantaneous memory and delayed memory scores.

## Orientation and calculation test

Orientation test includes telling the date (year, month, day), day of the week, and season of the year. The calculation capacity test required participants to do a serial subtraction of 7 starting from 100(up to 5 times). One point is obtained by answering one question correctly, with a total of 10 points.

## CESD-10

10 questions about mood, each with 4 answers. “Rarely or none of the time 1 day” =0, “some or a little of the time 1-2 days” =1, “Occasionally or a moderate amount of the time 3-4 days” =2, “Most or all of the time 5-7 days” =3. The higher the score, the worse the depression. The effectiveness of the CESD-10 in China’s elderly population has been proven.

## ADL, IADL

The ADL scale includes bathing or showering, eating, getting out of bed and walking, using the toilet and controlling urination or defection(rang0-6). The answers are divided into 4 levels: “no difficulty,” “have difficulty but can still do it," "have difficulty and need help," and "cannot do it." The IADL scale includes doing household, preparing hot meals, shopping, managing assets and taking medications(rang0-5). The answer items of both IADL and ADL scales are the same. In the subsequent analysis, we ordered the participants with the answer "no difficulty," to get 0 points, and the other answers to 1 point. And the final ADL score=6-total score, similarly, IADL=5-total score.

## Social and intellectual activities

Four kinds of social activities (interacting with friends; going to club; taking part in a community-related organization, and doing voluntary or charity) and four kinds of intellectual activities (playing mahjong, cards or chess; attending an educational or training course; investing in stock, and using the Internet) were used to assess activity intensity.

## Physical performance

The chair stand test refers to the time for the subject to stand up and sit five times continuously according to their usual rhythm without arm support, to evaluate the physical performance. Normal physical performance was defined as 5-time chair stand test <12 seconds, and low physical performance as 5-time chair stand test ≥12 seconds^42^. In addition, we also considered those who tried but failed to perform the chair standing test as low physical performance.

## Blood samples

Participants were asked to fast for one night, and then blood (three tubes) was collected by staff from the Chinese Center for Disease Control and Prevention (China CDC). Performing complete blood count (CBC) detection in CDC within two hours (stored and transported at 4°C). Blood samples were tested at the Youanmen Center for Clinical Laboratory of Capital Medical University (CMU). The samples are frozen at -20°C and transported to the Chinese CDC in Beijing within 2 weeks, where they are stored at -80°C until they are tested by CMU.

| Supplementary TablesSupplementary Table1 Basic characteristics of the participants in the training set and validation set | | | | | | |
| --- | --- | --- | --- | --- | --- | --- |
| **Characteristics †** | | **Total population** | | **Training set** | **Validation set** | ***P*** **‡** |
| **n** | | 5844 | | 2922 | 2922 |  |
| **Age** | 58.35(8.57) | | 58.28 (8.56) | | 58.42 (8.58) | 0.557 |
| **Sex=men (%)** | | 2690(46.0) | | 1368 (46.8) | 1322 (45.2) | 0.238 |
| **BMI-group=** | |  | |  |  | 0.05 |
| Low (%) | | 369(6.3) | | 199 (6.8) | 170 (5.8) |  |
| Normal (%) | | 3581(61.28) | | 1820 (62.3) | 1761 (60.3) |  |
| Overweight (%) | | 1606(27.48) | | 771 (26.4) | 835 (28.6) |  |
| Obesity (%) | | 288(4.93) | | 132 (4.5) | 156 (5.3) |  |
| **SBP (mmHg)** | | 129.31(20.67) | | 129.22 (20.93) | 129.39 (20.42) | 0.75 |
| **DBP (mmHg)** | | 75.38(11.83) | | 75.44 (12.03) | 75.32 (11.63) | 0.692 |
| **PP (mmHg)** | | 72.14(10.26) | | 72.12 (10.17) | 72.15 (10.35) | 0.928 |
| **WC(cm)** | | 84.16(12.66) | | 83.64 (12.89) | 84.67 (12.41) | 0.002 |
| **Handgrip strength (Kg)** | | 30.42(13.72) | | 30.56 (14.02) | 30.28 (13.42) | 0.445 |
| **Total Cognition Score** | | 10.53(4.16) | | 10.52 (4.20) | 10.54 (4.12) | 0.85 |
| **TICS** | | 6.58(2.81) | | 6.57 (2.82) | 6.60 (2.81) | 0.672 |
| **Orientation** | | 3.75(1.38) | | 3.74 (1.40) | 3.76 (1.36) | 0.538 |
| **Computation** | | 2.83(1.99) | | 2.83 (1.98) | 2.84 (1.99) | 0.864 |
| **Drawing ability** | | 0.64(0.48) | | 0.64 (0.48) | 0.64 (0.48) | 0.849 |
| **Episodic Memory** | | 3.31(1.87) | | 3.31 (1.88) | 3.30 (1.87) | 0.867 |
| **Immediate memory** | | 3.76(1.96) | | 3.76 (1.96) | 3.76 (1.95) | 0.995 |
| **Delayed memory** | | 2.86(2.04) | | 2.87 (2.05) | 2.85 (2.03) | 0.753 |
| **CESD-10** | | 9.85(4.85) | | 9.86 (4.88) | 9.83 (4.82) | 0.806 |
| **Nighttime sleep duration(hour)** | | 6.38(1.85) | | 6.33 (1.87) | 6.43 (1.82) | 0.041 |
| **Napping duration(minutes)** | | 32.64(43.18) | | 31.96 (43.19) | 33.31 (43.17) | 0.233 |
| **IADL** | | 0.31(0.78) | | 0.31 (0.79) | 0.31 (0.77) | 0.987 |
| **ADL** | | 2.43(1.41) | | 2.42 (1.40) | 2.44 (1.41) | 0.479 |
| **Social activity** | | 1.09(1.57) | | 1.06 (1.55) | 1.12 (1.58) | 0.195 |
| **Intellectual activity** | | 0.40(0.90) | | 0.40 (0.89) | 0.40 (0.91) | 0.896 |
| **Smoke=yes (%)** | | 2270(38.8) | | 1160 (39.7) | 1110 (38.0) | 0.188 |
| **Drink=** | |  | |  |  |  |
| often (%) | | 1513(25.9) | | 778 (26.6) | 735 (25.2) | 0.34 |
| sometimes (%) | | 459(7.9) | | 220 (7.5) | 239 (8.2) |  |
| never (%) | | 3872(66.3) | | 1924 (65.8) | 1948 (66.7) |  |
| **Physical performance=low (%)** | | 1671(28.6) | | 811 (27.8) | 860 (29.4) | 0.165 |
| **Medical history=** | |  | |  |  |  |
| hypertension (%) | | 1260(21.6) | | 617 (21.1) | 643 (22.0) | 0.426 |
| dyslipidemia (%) | | 583(10.0) | | 280 (9.6) | 303 (10.4) | 0.337 |
| lung disease (%) | | 543(9.3) | | 294 (10.1) | 249 (8.5) | 0.047 |
| liver disease (%) | | 269(4.6) | | 127 (4.3) | 142 (4.9) | 0.382 |
| heart disease (%) | | 617(10.6) | | 297 (10.2) | 320 (11.0) | 0.349 |
| kidney disease (%) | | 383(6.6) | | 176 (6.0) | 207 (7.1) | 0.113 |
| DM/HGlu (%) | | 320(5.5) | | 160 (5.5) | 160 (5.5) | 1 |
| digestive disease (%) | | 1203(20.6) | | 610 (20.9) | 593 (20.3) | 0.605 |
| ENP (%) | | 117(2.0) | | 60 (2.1) | 57 (2.0) | 0.852 |
| Memory disease (%) | | 76(1.3) | | 31 (1.1) | 45 (1.5) | 0.133 |
| Arth/Rheu (%) | | 1739(29.8) | | 861 (29.5) | 878 (30.0) | 0.647 |
| Asthma (%) | | 220(3.8) | | 115 (3.9) | 105 (3.6) | 0.536 |
| **Blood indexes** | |  | |  |  |  |
| WBC (G/L) | | 5.96[4.90,7.20] | | 5.90 [4.90, 7.20] | 6.00 [4.95, 7.20] | 0.658 |
| Hgb(g/dL) | | 14.20[13.00,15.50] | | 14.20 [13.03, 15.50] | 14.20 [13.00, 15.50] | 0.937 |
| MCV (fl) | | 91.30[86.90,95.60] | | 91.10 [86.70, 95.50] | 91.40 [87.00, 95.60] | 0.163 |
| PLT(G/L) | | 208.00[163.00,255.00] | | 207.00 [161.00, 254.00] | 208.00 [165.00, 258.00] | 0.184 |
| Crea (mg/dL) | | 0.76[0.64,0.87] | | 0.76 [0.64, 0.88] | 0.75 [0.64, 0.87] | 0.393 |
| BUN (mg/dL) | | 15.18[12.63,18.18] | | 15.14 [12.69, 18.01] | 15.24 [12.58, 18.34] | 0.343 |
| UA (mg/dL) | | 4.24[3.53,5.09] | | 4.23 [3.54, 5.06] | 4.26 [3.52, 5.10] | 0.426 |
| HbA1c (%) | | 5.10[4.90,5.40] | | 5.10 [4.90, 5.40] | 5.10 [4.90, 5.40] | 0.988 |
| hs-CRP (mg/L) | | 0.98[0.54,1.99] | | 0.99 [0.53, 2.00] | 0.98 [0.55, 1.98] | 0.52 |
| LDLC (mg/dL) | | 114.43[93.94,137.24] | | 114.43 [93.56, 136.86] | 114.82 [94.72, 137.63] | 0.606 |
| CHOL (mg/dL) | | 190.59[167.40,215.34] | | 190.59 [167.49, 215.34] | 190.79 [167.40, 214.56] | 0.819 |
| Glu(mg/dL) | | 102.24[94.32,112.68] | | 102.06 [94.32, 112.32] | 102.42 [94.50, 113.04] | 0.379 |
| TG (mg/dL) | | 104.43[74.34,152.44] | | 102.66 [74.34, 150.45] | 106.20 [75.22, 154.88] | 0.079 |
| HDLC (mg/dL) | | 49.48[40.59,59.92] | | 49.87 [40.98, 60.31] | 49.48 [40.59, 59.92] | 0.128 |
| TyG | | 4.64[4.46,4.86] | | 4.63 [4.45, 4.85] | 4.65 [4.46, 4.88] | 0.052 |
| AIP | | 0.74[0.27,1.26] | | 0.72 [0.26, 1.24] | 0.76 [0.28, 1.29] | 0.07 |

**Supplementary Tables**

**†** Quantitative data of normal and non-normal distribution were expressed as mean ± SD and medians with interquartile range (IQR). Categorical data were presented as amounts with percentages, respectively.

**‡** Comparisons of differences among groups were respectively analyzed by ANOVA test and Kruskal-Wallis test for continuous variables in line with normal and non-normal distribution, and by Chi-square test for categorical variables.

Abbreviations:

BMI Body Mass Index, SBP Systolic Blood Pressure, DBP Diastolic Blood Pressure, PP Pulse Pressure, WC waist circumference, TICS Telephone Interview of Cognitive Status, CESD-10 The 10-item Center for Epidemiological Studies, IADL Instrumental Activities of Daily Living, ADL Activities of Daily Living, DM/HGlu diabetes or high blood sugar, ENP Emotional, Nervous, or Psychiatric Problems, Arth/Rheu Arthritis or Rheumatism, WBC white blood cells, Hgb hemoglobin, MCV mean corpuscular, PLT platelet counts, Crea creatinine, BUN blood urea nitrogen, UA uric acid, HbA1c glycosylated hemoglobin, hsCRP high-sensitivity C reactive protein, LDLC low-density lipoprotein cholesterol, CHOL cholesterol, Glu glucose, TG triglycerides, HDLC high-density lipoprotein cholesterol, TyG triglyceride-glucose index, AIP atherogenic index of plasma

## Supplementary Table2 Variables with non-zero coefficients selected by Lasso Cox regression and the λ min based on the training set

| **Variables** | **Coefficients** | **λ†min** |
| --- | --- | --- |
| **Age** | 0.014 | 0.0057 |
| **SBP** | 0.016 |  |
| **PP** | 0.004 |  |
| **Low Physical Performance** | 0.154 |  |
| **Hypertension** | 0.033 |  |
| **Dyslipidemia** | 0.347 |  |
| **DM/HGlu** | 0.270 |  |
| **ENP** | 0.521 |  |
| **Memory Disease** | 0.433 |  |
| **CRP** | 0.018 |  |
| **CHOL** | 0.001 |  |
| **TyG** | 0.041 |  |
| **AIP** | 0.056 |  |

Fifty-one candidate variables were screened by Lasso cox regression. The variables initially included were: age, sex, BMI-group, SBP, DBP, PP, WC, Handgrip strength, Total Cognition Score, TICS, Orientation, Computation, Drawing ability, Episodic Memory, Delayed memory, CESD-10, Nighttime sleep duration, Napping duration, IADL, ADL, Social activity, Intellectual activity, smoke, drink, physical performance, hypertension, dyslipidemia, lung disease, liver disease, heart disease, kidney disease, DM/HGlu, digestive disease, ENP, Memory disease, Arth/Rheu, Asthma, WBC, Hgb, MCV, PLT, Crea, BUN, UA, HbA1c, hs-CRP, LDLC, CHOL,TyG, AIP

**†** The degree of LASSO regression complexity adjustment is controlled by the parameter λ. The larger the λ, the greater the penalty for the model with more variables. The smaller the λ, the smaller the error, and the better the performance of the model present.

Abbreviations could be seen in Supplementary Table 1.

## Supplementary Figures


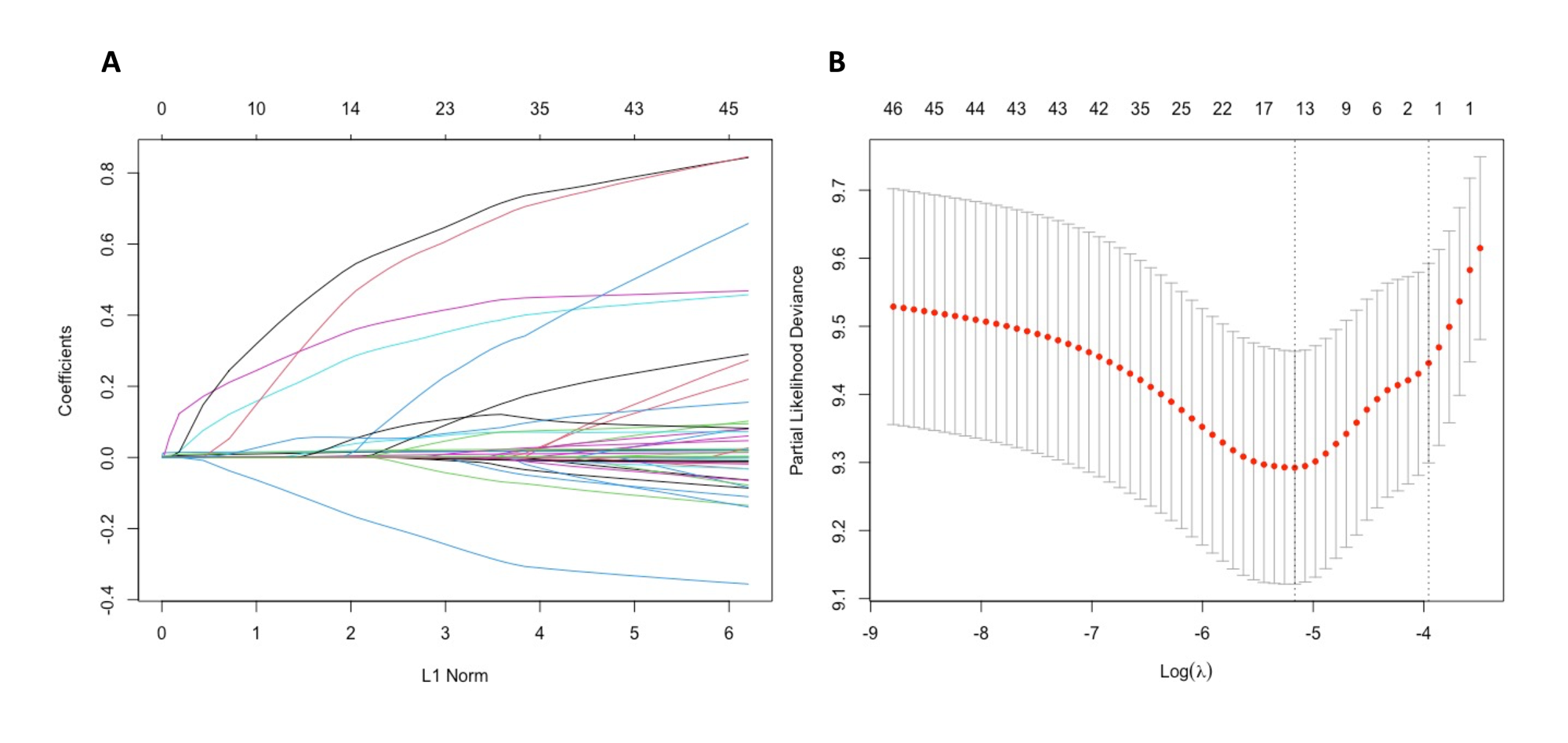


**Supplementary Figure1 Baseline characteristics selection for the predictors to new-onset stroke by Lasso COX regression model**

(A) LASSO coefficient profiles of the total of 50 variables in the model via penalized maximum likelihood with L1-norm regularization

(B) Hyperparameter of the LASSO regression model was performed by 10-fold cross-validation method, with the dotted vertical line was plotted at the optimal values using the minimum partial likelihood deviance


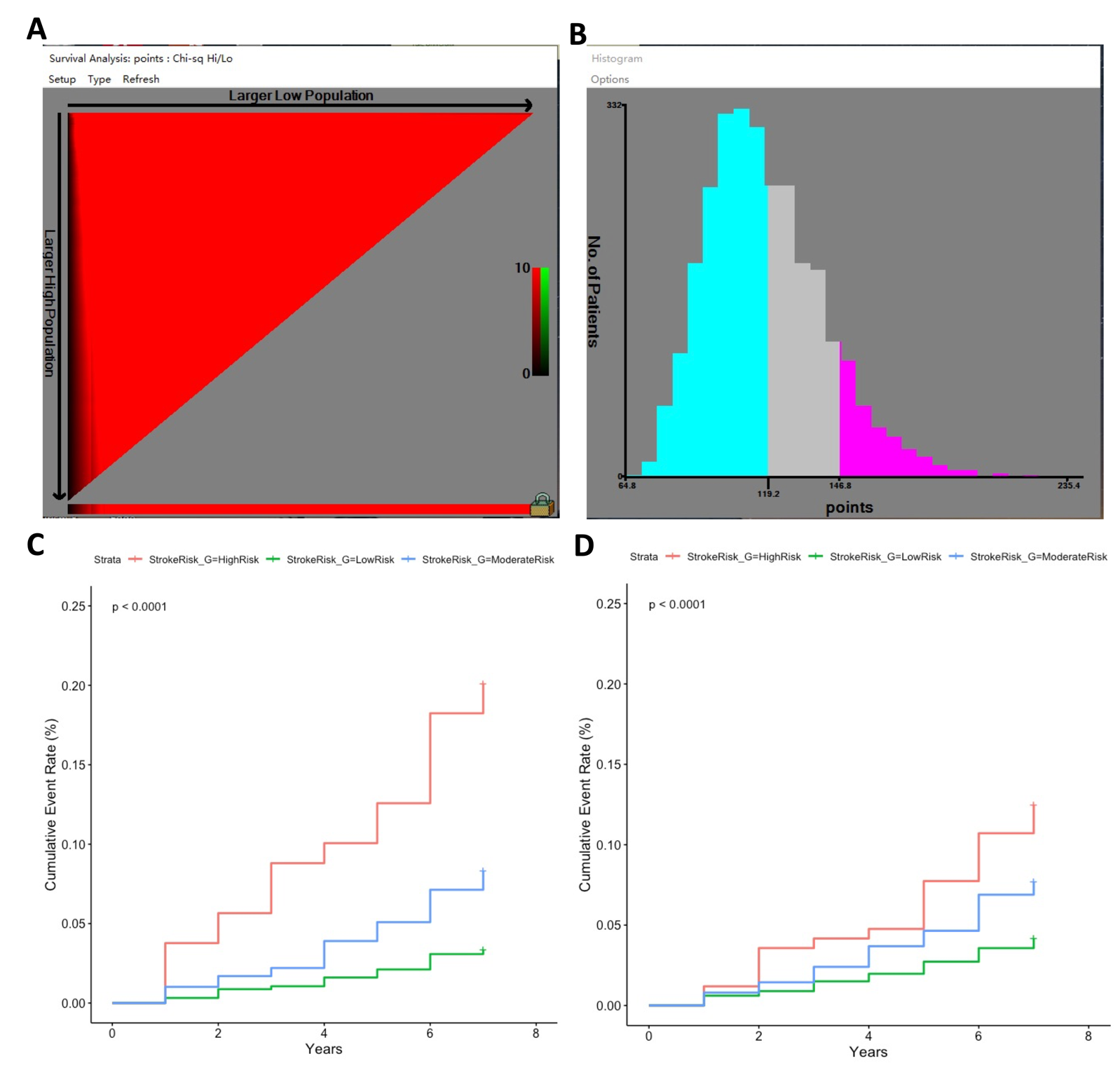


**Supplementary Figure2 Kaplan-Meier plots of cumulative incidence rate to new-onset stroke by log-rank test in Prediction Stratification Model constructed by X-tile program**

In the X-tile program(A), the points calculated by nomogram were cut off at the optional cut value, as determined by the significance of the plots (119.2, 146.8; p<0.001)

The population in the training set (B)and validation set(C) were respectively divided into low-moderate-high risk groups for the new-onset stroke by the cutoff value determined in the X-tile program. The cumulative incidence of the outcomes was significantly different by log-rank test.
